# Peer Review for “Checklist Approach to Developing and Implementing AI in Clinical Settings: Instrument Development Study”

**DOI:** 10.2196/69869

**Published:** 2025-02-20

**Authors:** Anonymous

**Keywords:** artificial intelligence, machine learning, algorithm, models, analytics, AI deployment, human-AI interaction, AI integration, checklist, clinical workflow, clinical setting, literature review

*This is the peer-review report for “Checklist Approach to Developing and Implementing AI in Clinical Settings: Instrument Development Study.”*

## Round 1 Review

The paper [1] presents the Clinical Artificial Intelligence (AI) Sociotechnical Framework (CASoF), a structured approach to guide the planning, design, development, and implementation of AI systems in health care settings. The framework is designed to address the gap between technical performance and sociotechnical factors that are essential for successful AI deployment in clinical environments.

The authors conducted a literature synthesis and a modified Delphi study involving global health care professionals to develop and refine the CASoF checklist. The checklist emphasizes the importance of considering the value proposition, data integrity, human-AI interaction, technical architecture, organizational culture, and ongoing support and monitoring, to ensure that AI tools are not only technologically sound but also practically viable and socially adaptable within clinical settings.

The study found that the successful integration of AI in health care depends on a balanced focus on both technological advancements and the sociotechnical environment of clinical settings. The CASoF represents a step forward in bridging this divide, offering a holistic approach to AI deployment that is mindful of the complexities of health care systems. The checklist aims to facilitate the development of AI tools that are effective, user-friendly, and seamlessly integrated into clinical workflows, ultimately enhancing patient care and health care outcomes.

The authors acknowledge some limitations of the study, such as the need for continuous refinement of the CASoF through iterative feedback and broader engagement with more stakeholders. Future research should aim to include an even wider array of perspectives, particularly from underrepresented regions and specialties, to enhance the framework’s comprehensiveness and applicability.

Overall, the paper provides a valuable contribution to the field of AI in health care by offering a practical and comprehensive approach to the development and implementation of AI systems in clinical settings.

## Abbreviations

AI: artificial intelligence
CASoF: Clinical Artificial Intelligence Sociotechnical Framework
